# Occupational Benzene Exposure and Risk of Male Genital Cancers: A Systematic Review and Meta‐Analysis

**DOI:** 10.1002/ajim.23740

**Published:** 2025-05-27

**Authors:** Alessandro Godono, Andrea Quattrocolo, Roberta Caradonna, Maria Vittoria Picciaiola, Paolo Boffetta, Monireh Sadat Seyyedsalehi

**Affiliations:** ^1^ Department of Public Health and Pediatrics University of Torino Turin Italy; ^2^ Department of Medical and Surgical Sciences University of Bologna Bologna Italy; ^3^ Stony Brook Cancer Center Stony Brook University Stony Brook New York USA; ^4^ Department of Family, Population and Preventive Medicine, Renaissance School of Medicine Stony Brook University Stony Brook New York USA

**Keywords:** benzene, male genital cancer, occupational exposure, occupational health, prostate cancer

## Abstract

**Background:**

Benzene is an established Group 1 carcinogen due to its leukemogenic properties. Recent studies suggest that occupational benzene exposure may be associated with solid cancers. However, little is known about its association with male genital cancers. We aimed to summarize the scientific evidence on occupational benzene exposure and the risk of male genital cancers.

**Methods:**

We searched for relevant articles in three electronic databases. Methodological quality and the certainty of evidence were evaluated using a modified version of the Newcastle‐Ottawa Scale (NOS) and Grading of Recommendations Assessment, Development and Evaluation (GRADE) assessment tool. We performed pooled and stratified meta‐analyses, as well as meta‐regressions to explore potential sources of heterogeneity.

**Results:**

Thirty‐one publications were included. Pooled results of incidence and mortality for prostate and testis cancer did not indicate a significant association with occupational benzene exposure. A borderline association was found for the incidence of prostate cancer (standardized incidence ratio (SIR): 1.07, 95% CI 0.97–1.19). Subgroup analyses stratified by study design and study quality revealed significant heterogeneity, with case‐control (relative risk (RR): 1.19, 95% CI 1.04–1.36) and high‐quality studies (RR: 1.22, 95% CI 1.14–1.31) showing an increased risk. Both NOS and GRADE assessments yielded mostly low to very low‐quality results.

**Conclusions:**

This review provides no clear evidence of an association between occupational exposure to benzene and the risk of male genital cancers. Subgroup analysis suggests an increased risk of prostate cancer in high‐quality studies. Nevertheless, it is important to acknowledge the methodological limitations of the available studies. Further analyses including methodologically sound studies are needed to corroborate these findings.

## 1 Background

Benzene is an aromatic hydrocarbon listed among high‐production‐volume chemicals by the Organization for Economic Cooperation and Development. It is generated in large quantities worldwide through catalytic reforming, cracking, or hydrodealkylation of toluene [1].

Currently, benzene is mainly found in the petroleum and chemical industries as an intermediate in the production of fuels, plastics, synthetic fibers, dyes, drugs, pesticides, and other commercial products. Other sources of human exposure to benzene include cigarette smoke, vehicle exhaust, and industrial emissions [2]. At present, due to the identification of its carcinogenic properties, its use has decreased significantly, and it is mainly employed to synthesize other aromatic compounds and for octane enhancement of unleaded gasoline [3].

Occupational exposure to benzene primarily affects the hematopoietic and the respiratory systems. Short‐term effects include drowsiness, dizziness, tremors, headache, confusion, loss of consciousness [4], and irritation of the skin, eyes, and respiratory tract [5]. Long‐term effects are mainly related to its hematotoxic and immunosuppressive properties [5]. In 1979, the International Agency for Research on Cancer (IARC) classified benzene as a human carcinogen based on sufficient evidence that it causes leukemia [6]. This assessment was confirmed in 2018 specifically for acute myeloid leukemia and acute nonlymphoid leukemia [6].

The association between the risk of solid tumors and occupational benzene exposure is still under investigation. Recent meta‐analyses of available evidence found a positive association between occupational benzene exposure and several solid tumors, including breast [7], lung [8], bladder [9], kidney [9], colorectal [10], nervous system [11], and head and neck cancers [12]. However, to our knowledge, no similar studies have been conducted on male genital cancers.

The scientific literature on male genital cancers is limited and inconsistent. Among these cancers, prostate cancer represents more than 90% of all male genital cancers, and is one of the most diagnosed cancers worldwide, globally in 2022 the fourth most frequently diagnosed malignancy according to the World Health Organization (WHO) [13]. According to data from EUROCARE‐5, the 5‐year relative survival rate for prostate cancer in Europe is 83% overall, and 90% for men diagnosed between 55 and 64 years of age [14]. In 2022, its mortality rates varied between 3.1 cases per 100,000 men in South‐Central Asia and 29.7 in Southern Africa [15]. Its incidence also varies widely among countries, and it is greatly influenced by prostate‐specific antigen‐based screening programs. Europe had the highest incidence rate in 2022 (82.8 per 100,000 men). The world mortality‐to‐incidence ratio for prostate cancer computed from the GLOBOCAN 2012 database was 28.1% with high geographic variability [16] Moreover, research indicates that there are differences in prostate cancer susceptibility among different ethnic groups [17]. The etiology of prostate cancer can be considered multifactorial, likely resulting from the interaction between genetic patterns and other modifiable risk factors such as hormone levels, sexual behavior, and chronic inflammation. Also, environmental and occupational exposures have been suggested as possible risk factors for this disease, without definite evidence for any agent [18].

Other male genital cancers include testicular and penile cancer, accounting together approximately for 1.5% of all male genital cancers [19]. Several hormonal and environmental factors have been hypothesized as related to testicular carcinogenesis, but the only factors clearly associated with testicular cancer are family history of testicular cancer, prior unilateral testicular cancer, and congenital abnormalities [20]. For penile cancer, several risk factors are known including human papilloma virus, history of chronic inflammation, penile trauma, lack of neonatal circumcision, tobacco use, balanitis xerotica obliterans, poor genital hygiene, and a history of sexually transmitted infections [21]. Some occupational and environmental pollutants such as bisphenol A, phthalates, metals, polychlorinated biphenyls, and organochlorines are hypothesized to act as endocrine‐disrupting chemicals, leading to testicular and penile cancer, but the evidence is not conclusive [22]. Data on benzene endocrine activity is still controversial and under debate [23].

Therefore, we aimed to perform a systematic review and meta‐analysis of occupational studies to address the potential association between benzene exposure and the occurrence of genital tract cancers, with a particular focus on prostate cancer.

## 2 Methods

### 2.1 Search Strategy

The research protocol used for this Meta‐Analysis and Systematic Review was registered in the PROSPERO Database under Registration Number CRD42022379720.

This study was conducted in agreement with the Conducting Systematic Reviews and Meta‐Analysis for Observational Studies of Etiology guide [24] and the Preferred Reporting Items for Systematic Reviews and Meta‐Analyses statement (Supporting Information S1: Tables 1A, 1B) [25].

We included all studies mentioned in the most recent IARC monograph on benzene (IARC, 2018) and we performed a systematic search for articles on the association between benzene exposure and the risk of any solid cancer in three relevant electronic databases: EMBASE (Ovid), MEDLINE (PubMed), SCOPUS, from their inception to April 2024.

Supporting Information S1: Table 2 reported the complete search string. In this analysis we included the articles that investigated the correlation between occupational exposure to benzene and male genital cancer (prostate, testis, and other male genital cancers), with either cancer mortality or incidence as endpoints.

### 2.2 Eligibility Criteria

Two reviewers (M.S.S., A.Q.) reviewed independently the list of titles, abstracts, and full text of articles identified in the search. Disagreement was solved by consensus involving a third reviewer (A.G.).

The selection process included the following steps: 1) scanning of title and abstract of articles; 2) reviewing the full texts of articles included after the first screening; 3) reviewing the references of articles included after the second step. The inclusion criteria considered in the meta‐analysis were:

1. Study design: cohort studies, nested case‐control studies, community‐based case‐control studies, and hospital‐based case‐control studies.
2. Exposure: employment in occupations in which benzene occurs as a significant or primary source of exposure, including the petroleum industry, rubber industry, chemical industry, shoemaking, paint production and painting, printing, and laboratories or occupations with ascertained benzene exposure evaluation.
3. Outcome: studies that report risk measures for male genital cancers, including odds ratios (OR), standardized mortality ratios (SMR), standardized incidence ratios (SIR), risk ratios or relative risks (RR), and hazard ratios (HR), along with 95% confidence intervals (CI) or that provide adequate data for computational analysis.

The criteria for exclusion were:

1. Exposure: studies focusing on workers primarily exposed to other carcinogenic agents such as PAH, asbestos, silica, or butadiene.
2. Type of cancer: studies reporting results for cancers not related to male genital tract.
3. Language: studies written in languages other than English, Spanish, French, German, or Italian.

Inclusion and exclusion criteria are summarized in Supporting Information S1: Table 3 following the PECOS methodology [26].

### 2.3 Data Extraction

After removing duplicates and assessing eligibility, three reviewers (M.S.S., A.Q., R.C.) independently extracted relevant data from the included papers. Any disagreements were resolved with the involvement of a fourth reviewer (A.G.).

For each study, RRs, SMRs, and SIRs for cohort studies and ORs for case‐control studies, along with their 95% confidence intervals (95% CI), were extracted as outcome estimates. When articles did not report risk estimates and their respective 95% CIs, they were calculated through the number of observed and expected cases. If 90% CIs were reported, they were converted to 95% CIs.

Additional study variables were also extracted, if reported in the article: name of first author, year of publication, location, type of study, type of industry, period of occupational exposure, duration of follow‐up, duration of employment, confounders, cancer site (prostate cancer, testis cancer and unspecified male genital cancers), and outcome measures.

### 2.4 Quality Assessment

Quality assessment was performed independently by three reviewers (M.S.S., A.Q., M.V.P.) through a modified version of the Newcastle‐Ottawa Scale (NOS) [27]. The following additional item was introduced to account for the exposure‐response criterion: “Did the study evaluate an exposure‐response relationship based on estimated levels of benzene exposure for all individuals?”. Studies were penalized if they did not rely on estimated exposure levels, but instead used exposure proxies that may not accurately reflect the true level of exposure. A total of 11 criteria were applied to cohort studies and 10 criteria to case‐control studies; each met criterion was assigned one or two points, with the exception of the exposure‐response criterion, which deducted 2 points for studies based on exposure proxies (e.g., ever worked, ever exposed, work duration, duration of exposure) that failed to provide an estimated level of exposure‐response gradient. During this evaluation procedure another author (AG) was consulted in case of divergence among the reviewers. Since no predefined cut‐offs are provided in the NOS score, the Authors considered studies with a score ≥ 8 as high quality, moderate quality studies with scores 6–7 and low quality studies those with scores < 6. The complete list of NOS quality assessment criteria is provided in Supporting Information S1: Table 4.

### 2.5 Quality of Evidence Assessment

The Grading of Recommendations Assessment, Development and Evaluation (GRADE) framework [28] was employed to evaluate the overall certainty of evidence across studies for each outcome. This framework classifies the overall quality of evidence into four classes: “high,” “moderate,” “low,” and “very low.” In line with GRADE methodology, the certainty of evidence is initially rated as low due to the observational nature of the included studies. Later, the certainty of evidence can be upgraded or downgraded considering the following GRADE domains. Upgrading reasons include: 1. Large magnitude of effect size; 2. Presence of plausible residual confounding that may increase the magnitude of the effect; 3. Exposure‐response gradient. Downgrading reasons consider: 1. Limitations in studies; 2. Four types of Indirectness (population, intervention, outcomes, indirect comparison); 3. Inconsistency; 4. Imprecision; 5. Publication bias. While GRADE offers a systematic and transparent approach to rating the certainty of evidence, some limitations of the GRADE system should be acknowledged, particularly when applied to occupational epidemiology. The default assumption that observational studies start as low‐certainty may not fully reflect their methodological strengths, especially in settings where randomized trials are not feasible. Additionally, the framework does not formally incorporate key elements of causal inference relevant in occupational settings, such as biological plausibility and mechanistic evidence that may strengthen the interpretation of findings. These evaluations were performed by two independent reviewers (M.V.P., M.S.S.); discrepancies were resolved with the help of a third reviewer (A.G.).

### 2.6 Statistical Analysis

We estimated pooled RR and the corresponding 95% confidence intervals (CIs) of the association between male genital cancer and benzene exposure through a series of random effects meta‐analyses, both combining and separating results for incidence and mortality. When both estimates were available, we utilized the data on incidence for the combined pooled estimate. The restricted maximum likelihood estimator [29] was used to calculate the heterogeneity variance τ^2^. We used Knapp‐Hartung adjustments [30] to calculate the CI around the pooled effect. We evaluated heterogeneity using the Cochran's Q test and I² statistic. Heterogeneity was considered low for values of *I* ^2^ < 30%, moderate between 30% and 59%, and high for values ≥ 60%.

Moreover, we conducted stratified meta‐analyses of prostate and testis cancer by outcome (incidence, mortality), study design (cohort studies, case‐control studies), type of industry (oil industry, chemical industry, other industry, unspecified industry), geographic region (European countries North American countries, other countries) and study quality (high, intermediate, low).

Meta‐regression analyses were also performed to test the association between duration of employment, duration of follow‐up, quality‐assessment evaluated with the NOS score, and male genital cancers RRs.

To evaluate the stability of results, sensitivity analyses were conducted, repeating the main meta‐analysis after excluding one study at a time to assess individual influence on the overall results.

Finally, we assessed the presence of publication bias by visual inspection of the funnel plot and applying the regression asymmetry test proposed by Egger et al. [31].

## 3 Results

**Figure 1:**
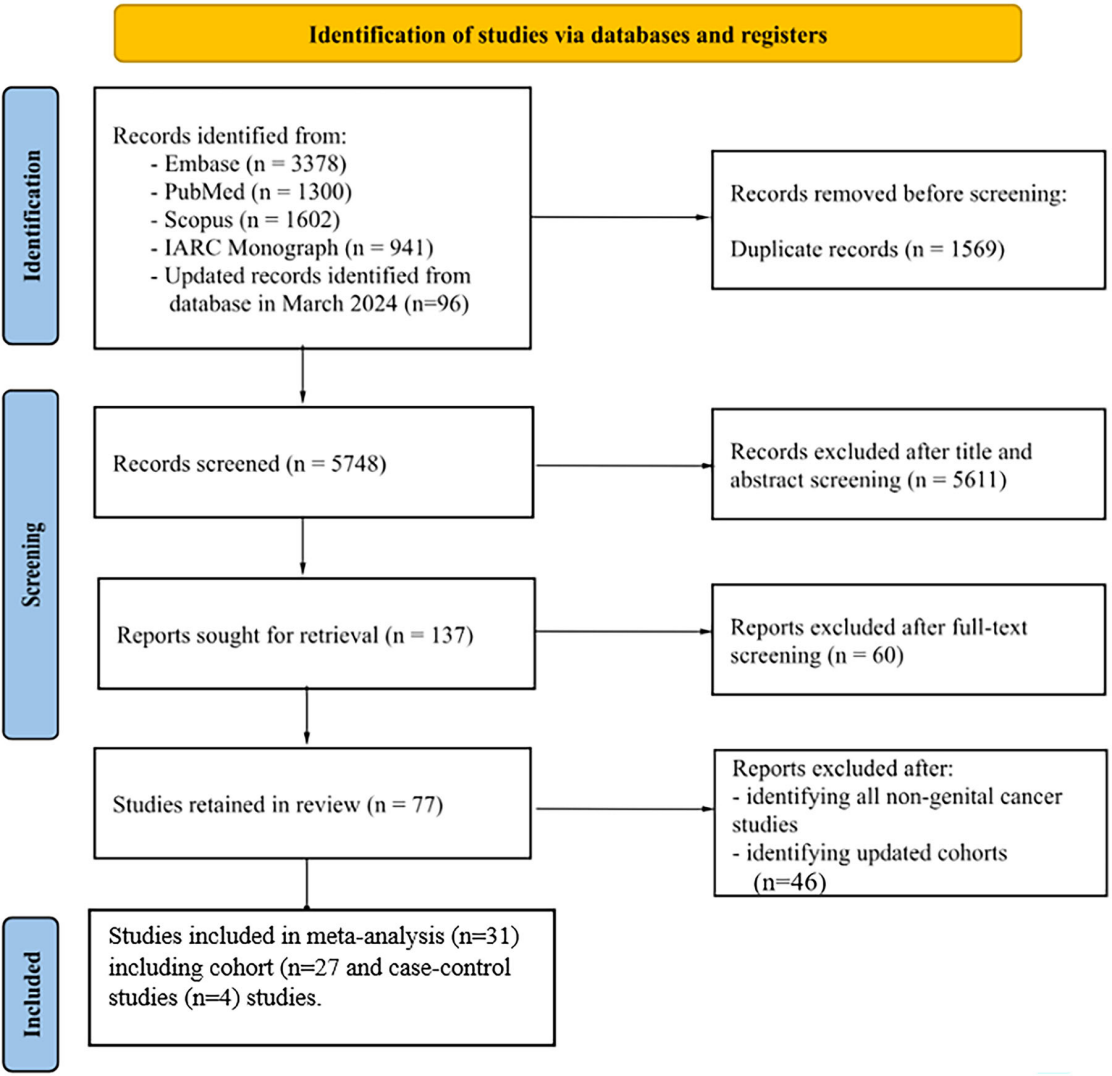
Selection of studies for inclusion in the review and meta‐analysis.

**Figure 2:**
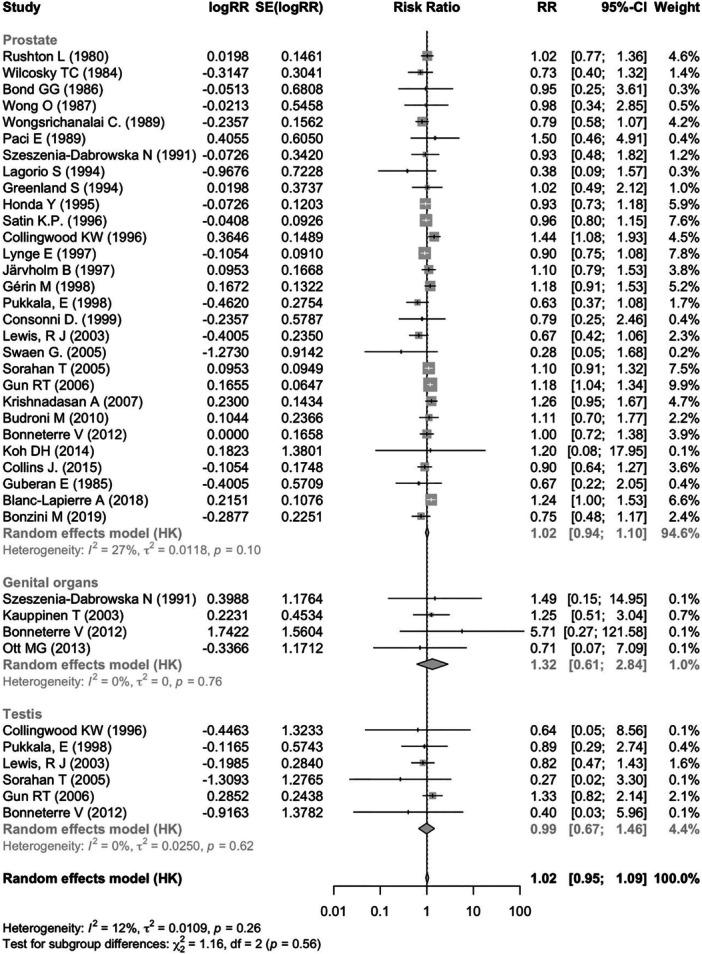
Forest plot (random‐effects model) of results on the association between benzene exposure and male genital cancer.

The literature search combined with studies extracted from the 2018 IARC monograph, returned 7317 articles. After the removal of duplicates (*n* = 1569), 5748 articles remained. After reviewing the titles and abstracts, 137 were considered relevant for inclusion. The full texts were examined and assessed against exclusion and inclusion criteria. Sixty articles did not meet the eligibility criteria. Finally, after the exclusion of studies not reporting results of male genital cancers studies and earlier reports of included cohorts (*n* = 46), a total of 31 publications were retained [32, 33, 34, 35, 36, 37, 38, 39, 40, 41, 42, 43, 44, 45, 46, 47, 48, 49, 50, 51, 52, 53, 54, 55, 56, 57, 58, 59, 60, 61, 62] comprising 26 cohort studies and 5 case‐control studies (Figure 1). No hospital‐based study was retrieved. Details of the search and selection of studies are provided in Table 1. Included articles were published from 1980 [58] to 2019 [46]. Most cohort studies were conducted in Europe (*n* = 15), and North America (*n* = 14). The industry most frequently studied was the oil refinery industry (*n* = 16), followed by the chemical industry (*n* = 4). We extracted 29 estimates for prostate cancer, 6 for testicular cancer, and 4 estimates referring to male genital organs more generally. Estimates for penile cancer were not available in the included studies. The overall RR of prostate cancer for benzene exposure was 1.02 (95% CI: 0.94–1.10; *I* ^2^: 27%; p‐het: 0.100); that for testis cancer was 0.99 (95% CI: 0.67–1.463; *I* ^2^: 0%; p‐het: 0.620) and that for overall genital cancer was 1.32 (95% CI: 0.61–2.84; *I* ^2^: 0%; p‐het: 0.760) (Figure 2). After stratification by outcome, studies reporting results on cancer incidence yielded an overall SIR of 1.07 (95% CI: 0.97–1.19) for prostate cancer and an overall SIR of 1.00 (95% CI: 0.63–1.58) for testis cancer. For studies reporting mortality results, the overall SMR was 0.95 (95% CI: 0.87–1.04) for prostate cancer and 1.46 (95% CI: 0.35–4.95) for testis cancer.

**Table 1:** Selected characteristics of the studies included in the review and meta‐analysis.

ID	Study	Country	Study type	Industry	Cancer type	Outcome	Observed	RR	Lower 95CI	Upper 95CI	NOS score	Adj NOS
1	Budroni M. 2010	Italy	Cohort	Petrochemical	Prostate	Incidence	18	1.11	0.7	1.77	6	4
2	Satin K.P. 1996	USA	Cohort	Oil refinery	Prostate	Mortality	/	0.96	0.8	1.15	6.5	4.5
3	Greenland S. 1994	USA	Case control	Transformer‐assembly facility	Prostate	Mortality	/	1.02	0.49	2.12	6	4
4	Bond G.G. 1986	USA	Cohort	Chemical	Prostate	Mortality	3	0.95	0.19	2.74	8	6
5	Collingwood K.W. 1996	USA	Cohort	Oil refinery	Prostate	Mortality	49	1.44	1.06	1.9	7	5
					Testis	Mortality	1	0.64	0.02	3.58	7	5
6	Honda Y. 1995	USA	Cohort	Petroleum Manufacturing Plant	Prostate	Mortality	74	0.93	0.73	1.17	8	6
7	Paci E. 1989	Italy	Cohort	Shoe workers	Prostate	Mortality	3	1.5	0.38	4.08	8	6
8	Gérin M. 1998	Montreal	Case control	Unspecified	Prostate	Incidence	95	1.18	0.91	1.53	8	8.5
9	Pukkala, E. 1998	Finland	Cohort	Oil refinery	Prostate	Incidence	15	0.63	0.35	1.03	7	5
					Testis	Incidence	4	0.89	0.24	2.28	7	5
10	Koh D.H. 2014	Korea	Cohort	Refinery/petrochemical complex	Prostate	Mortality	1	2.51	0.06	14	6	4
						Incidence	1	1.2	0.03	6.71	6	4
11	Bonneterre V. 2012	France	Cohort	Chlorochemical plant	Prostate	Incidence	40	1	0.71	1.36	7.5	5.5
					Testis	Incidence	1	0.4	0.01	2.22	7.5	5.5
					Genital organs	Incidence	1	5.71	0.07	31.74	7.5	5.5
12	Järvholm B. 1997	Sweden	Cohort	Oil refinery	Prostate	Incidence	68	1.10	0.79	1.53	8	6
13	Szeszenia‐Dabrowska N. 1991	Poland	Cohort	Rubber workers	Prostate	Mortality	9	0.93	0.45	1.72	6	4
					Genital organs	Mortality	1	1.49	0.07	7.04	6	4
14	Wilcosky T.C. 1984	USA	Case‐control	Rubber workers	Prostate	Mortality	11	0.73	0.38	1.27	5.5	3.5
15	Bonzini M. 2019	Italy	Cohort	Oil refinery	Prostate	Mortality	20	0.75	0.48	1.16	7	5
16	Sorahan T. 2005	UK	Cohort	Benzene‐exposed workers	Prostate	Mortality	50	0.94	0.7	1.24	7.5	5.5
						Incidence	121	1.1	0.91	1.32	7.5	5.5
					Testis	Mortality	0	0	0	3.37	7.5	5.5
						Incidence	1	0.27	0.01	1.49	7.5	5.5
17	Kauppinen T. 2003	Finland	Cohort	Chemical laboratory	Genital organs	Incidence	6	1.25	0.46	2.72	8	6
18	Ott M.G. 1978	USA	Cohort	Occupational exposed	Genital organs	Mortality	1	0.71	0.03	3.52	7	5
19	Guberan E. 1985	Switzerland	Cohort	Perfumery and flavor industry	Prostate	Mortality	4	0.67	0.16	1.5	8	6
						Incidence	5	0.74	0.24	1.7	8	6
20	Lewis, R.J. 2003	Canada	Cohort	Petroleum workers	Prostate	Mortality	1	0.21	0.01	1.07	7	5
						Incidence	20	0.67	0.41	1.03	7	5
					Testis	Mortality	3	1.86	0.47	5.07	7	5
						Incidence	14	0.82	0.45	1.37	7	5
21	Wongsrichanalai C. 1989	USA	Cohort	Petroleum refinery	Prostate	Mortality	44	0.79	0.58	1.07	8	6
22	Lagorio S. 1994	italy	Cohort	Service station workers	Prostate	Mortality	2	0.38	0.07	1.19	7	5
23	Consonni D. 1999	Italy	Cohort	Oil refinery	Prostate	Mortality	4	0.79	0.21	2.03	8	6
24	Krishnadasan A. 2007	USA	Case control	Aerospace radiation workers	Prostate	Incidence	42	1.26	0.95	1.67	8.5	8.5
25	Blanc‐Lapierre A 2018	Canada	Case control	Unspecified	Prostate	Incidence	231	1.24	1.01	1.54	8.5	8
26	Lynge E. 1997	Europe	Cohort	Service station workers	Prostate	Incidence	127	0,9	0.7	1	7	5
27	Rushton L. 1980	UK	Cohort	Oil refinery	Prostate	Mortality	47	1.02	0.76	1.35	7	5
28	Gun R.T. 2006	Australia	Cohort	Petroleum industry	Prostate	Incidence	251	1.18	1.04	1.34	8	6
						Mortality	35	0.94	0.65	1.3	8	6
					Testis	Incidence	19	1.33	0.8	2.08	8	6
						Mortality	1	1.01	0.03	5.63	8	6
29	Wong O. 1987	USA	Cohort	Chemical	Prostate	Mortality	/	0.98	0.34	2.85	8	8
30	Swaen G. 2005	Netherlands	Cohort	Chemical	Prostate	Mortality	/	0.28	0.04	0.44	8	6
31	Collins J. 2015	USA	Cohort	Refinery	Prostate	Mortality	/	0.90	0.63	1.25	7.5	5.5

Subgroup analyses of prostate and testis cancer, stratified by geographic region and type of industry did not highlight heterogeneity. However, significant heterogeneity was observed in subgroup analysis of prostate cancer by study design (*p* = 0.005) and adjusted study quality (*p* = 0.007). Specifically, case‐control studies showed an increased RR of prostate cancer (1.19; 95% CI: 1.04–1.36), compared to cohort studies (1.01; 95% CI: 0.95–1.08) while high‐quality studies (according to the adjusted NOS score) showed an increased prostate cancer RR (1.22; 95% CI 1.14–1.31) compared to lower quality studies. Table 2 summarizes the results of stratified meta‐analyses.

**Table 2:** Results of metanalyses by type of cancer, region, outcome, study design, and type of industry.

	Prostate cancer				Testis cancer			
Characteristic	No. of studies	RR (95% CI)	*I* ^2^ (*p*‐value)	*P*_het_ *	No. of studies	RR (95% CI)	*I* ^2^ (*p*‐value)	*P*_het_ *
**Outcome**								
Incidence	14	1.07 (0.97, 1.19)	29.8% (0.14)	0.06	5	1.00 (0.63, 1.58)	0.0% (0.50)	0.24
Mortality	21	0.95 (0.87, 1.04)	0.0% (0.59)		3	1.46 (0.43, 4.95)	0.0% (0.73)	
**Region**								
North America	13	1.02 (0.90, 1.19)	35.3% (0.10)	0.31	2	0.81 (0.43, 1.55)	0.0% (0.46)	0.11
Europe	14	0.97 (0.87, 1.07)	0.0% (0.73)		3	0.68 (0.17, 2.75)	0.0% (0.59)	
Other	2	1.18 (1.16, 1.19)	0.0% (0.48)		1	1.33 (0.82, 2.14)		
**Type of industry**								
Oil industry	16	0.96 (0.86, 1.08)	44.3% (0.25)	0.12	4	1.04 (0.67; 1.62)	0.0% (0.73)	0.46
Chemical industry	3	1.00 (0.96, 1.03)	0.0% (0.99)		1	0.40 (0.93, 5.96)		
Other industries	7	1.13 (0.95, 1.34)	0.0% (0.65)		0			
Unspecified	3	1.15 (0.78, 1.68)	35.4% (0.10)		1	0.27 (0.02; 3.29)		
**Study design**								
Cohort study	24	1.01 (0.95, 1.08)	25.1% (0.58)	0.01	5	0.98 (0.67, 1.45)	0.0% (0.80)	—
Case‐control study	5	1.19 (1.04, 1.36)	0.0% (0.54)		0			
**Study quality**								
Low	1	0.73 (0.40; 1.32)	0.0% (0.49)	0.12	0			0.63
Intermediate	12	0.94 (0.80; 1.10)	31.4% (0.14)		2	0.83 (0.55; 1.26)	0.0% (0.49)	
High	16	1.08 (0.99; 1.18)	2.3% (0.426)		4	0.97 (0.35; 2.69)	0.0% (0.80)	
**Adjusted study quality**								
Low	16	0.96 (0.87; 1.08)	21.5% (0.33)	0.01	2	0.83 (0.55; 1.26)	0.0% (0.49)	0.64
Intermediate	9	1.00 (0.84; 1.18)	26.50% (0.74)		4	0.97 (0.35; 2.69)	0.0% (0.80)	
High	4	1.22 (1.14; 1.31)	0.0% (0.96)		0			

* *P*_het_: *p*‐value for heterogeneity between subgroups using Cochran's *Q* test.

**Figure 3:**
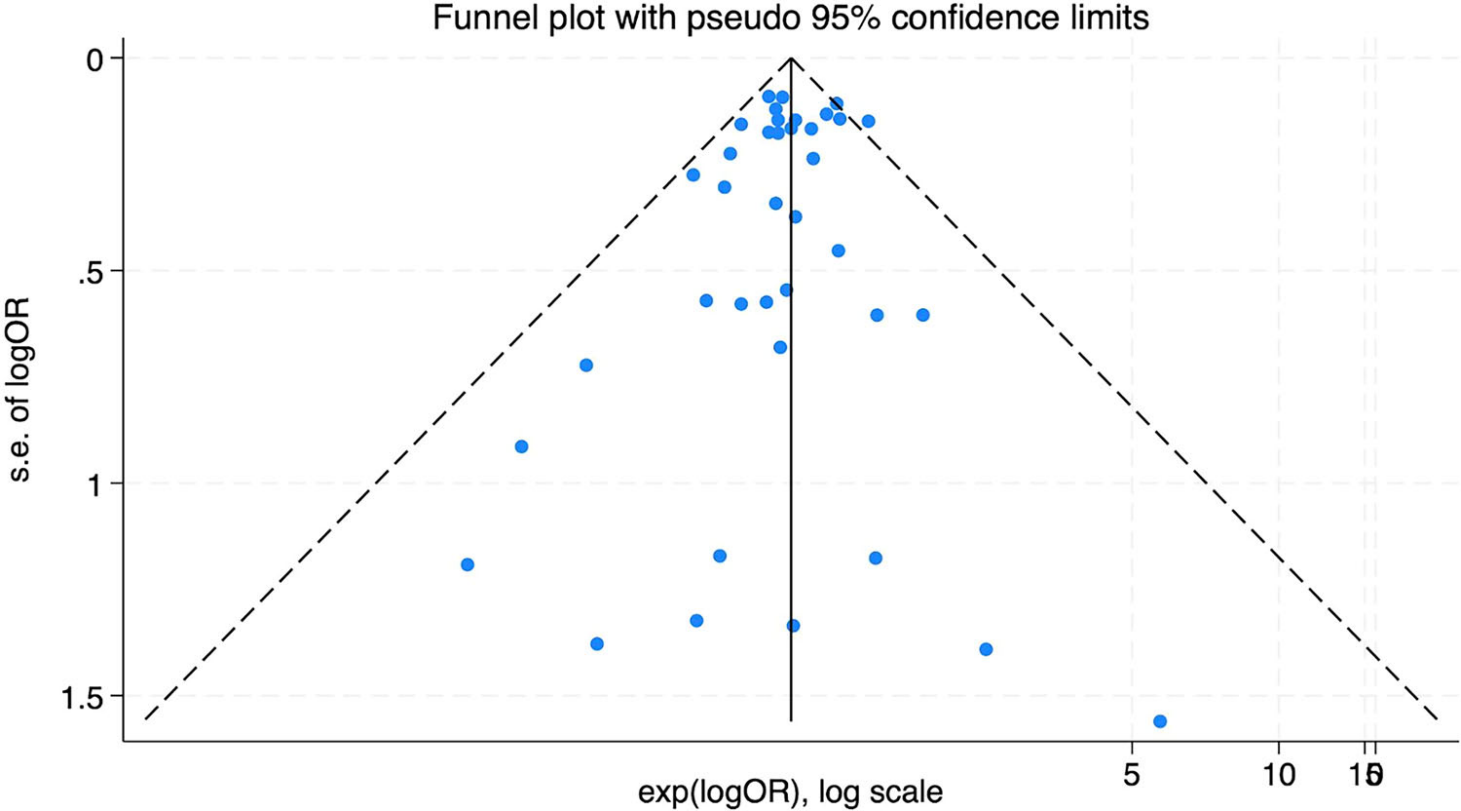
Funnel plot of results on the association between benzene exposure and overall risk of male genital cancers.

Repeating the meta‐analyses after the omission of any single article from the main meta‐analysis did not influence pooled RRs which ranged from 0.98 (95% CI: 0.91–1.05) when excluding the study by Blanc‐Lapierre A. et al. [56], to 1.01 (95% CI: 0.94–1.09) when excluding the study by Wongsrichanalai [52]. The overall Egger test (*p* = 0.332) and the funnel plot examination indicated no evidence of publication bias (Figure 3).

The results of the meta‐regressions indicated a positive association between quality assessment adjusted score and risk of prostate cancer in workers occupationally exposed to benzene (*p* = 0.012), suggesting that higher quality studies obtained larger effect sizes. No association was found with duration of employment (*p* = 0.980), the duration of follow‐up (*p* = 0.110) and the unadjusted NOS score (*p* = 0.090) (Supporting Information S1: Figure 1). The results of quality assessment are presented in Supplementary Table 5. According to the original NOS score, 16 studies were classified as high quality, 14 as moderate quality and 1 as low quality, while the adjusted version of the NOS score classified only 4 studies as high quality, 10 as moderate and 17 as low quality. Table 3 summarizes the quality of evidence rating according to the GRADE system. All pooled estimates received a rating of “very low” quality due to risk of bias or imprecision, while the estimates for overall testis cancer, testis cancer mortality, and overall genital organ cancer were rated “very low” due to both risk of bias and imprecision. The full descriptive justification is available in Supporting Information S1: Table 6.

**Table 3:** GRADE summary of findings.

Outcome	Number of included studies	GRADE	RR (95% CI)
Overall RR for prostate cancer	29	Very low (due to risk of bias)	1.02 (0.94, 1.10)
SMR for prostate cancer	21	Very low (due to risk of bias)	0.95 (0.87, 1.04)
SIR for prostate cancer	14	Very low (due to risk of bias)	1.07 (0.97, 1.19)
Overall RR for testis cancer	6	Very low (due to risk of bias)	0.99 (0.67, 1.46)
SMR for testis cancer	3	Very low (due to risk of bias and imprecision)	1.46 (0.93, 4.95)
SIR for testis cancer	5	Very low (due to risk of bias)	1.02 (0.63, 1.58)
Overall RR for male genital cancer	4	Very low (due to risk of bias and imprecision)	1.32 (0.61, 2.84)

Abbreviations: RR, relative risk; SMR, standardized mortality ratio; SIR, standardized incidence ratio.

## 4 Discussion

This systematic review and meta‐analysis summarized the evidence on the association between occupational exposure to benzene and the risk of male genital cancers. No association emerged, with no heterogeneity observed according to outcome, type of industry, and geographic region. There was an indication of heterogeneity in the subgroup analysis by study design and adjusted study quality, with case‐control studies and high‐quality studies showing an increased risk of prostate cancer compared to the other subgroups. We acknowledge that case‐control studies may be generally more susceptible to biases such as selection bias, which might have influenced the results in the overall summary estimate. Nevertheless, the case‐control studies included in the analysis had an overall better quality compared to the cohort studies. Among the four high‐quality studies classified according to the adjusted NOS score, three of them were case‐control studies that considered benzene concentration measurements and provided exposure‐response estimates of risk. In contrast, all other studies, except for one cohort study [60], relied on different proxies of exposure, such as duration of exposure or employment, or did not account for exposure levels at all, considering only whether individuals had ever been exposed to or employed in a benzene‐using industry. This approach inevitably includes workers who were not actually exposed or individuals with low exposure levels due to different job tasks, leading to exposure misclassification. Since such misclassification—at least in cohort studies—is likely to have been non‐differential with respect to the outcome of interest, it is plausible that the resulting bias was toward the null. Hence, the overall findings may reflect the methodological limitations of the studies included rather than an actual absence of association. It is important to note, though, that results from moderator analyses should be interpreted with caution, as the potential for spurious findings increases due to multiple testing.

To date, there is strong evidence for genotoxic effects of benzene metabolites on haematopoietic stem cells, linking its occupational exposure to the development of leukemia, particularly acute myeloid leukemia. Its genotoxic metabolism is inherently complex, targeting critical genes and pathways through the induction of genetic, chromosomal, or epigenetic abnormalities; stromal cell dysregulation; dysregulated apoptosis of hematopoietic stem cells (HSCs) and stromal cells; and altered proliferation and differentiation of HSCs [63].

By contrast, less is known about benzene exposure and the risk of solid cancers. Recent systematic reviews and meta‐analyses suggested that workers with occupational exposure to benzene might be at increased risk of breast [7], lung [8], bladder [9], kidney [9], colorectal [10], nervous system [11], and head and neck cancers [12]. The etiology of male genital cancers is multifactorial and quite elusive, with modifiable and unmodifiable risk factors associated with their development. The only certain risk factors for prostate cancers are related to age, familial history, and African ancestry, while genetic factors can explain only a portion of familial cancers [64]. Nevertheless, some epidemiological evidence [65] suggests a role of environmental exposures including obesity, sedentary lifestyle, alcohol consumption, diet, night shift work, and certain pesticides. However, the biological plausibility of the involvement of certain chemicals in prostate carcinogenicity has not been fully clarified. Some hypotheses are derived from the results of experimental animal studies considering hormone‐mediated pathway and the disruption of normal enzymatic activities and immune system functions [66].

Our results confirm this uncertainty in the association between benzene exposure and the risk of male genital cancers.

This systematic review and meta‐analysis is, to our knowledge, the first to address the association between benzene exposure and the risk of male genital cancers. We adopted an extensive search strategy and a rigorous approach to include moderate‐ and high‐quality cohort and case‐control studies. However, the overall quality of the available studies, as evaluated through the adjusted NOS, was found to be generally moderate to low, which significantly limits the robustness of our findings. Despite being widely used and validated in various settings, the NOS rating system has inherent weaknesses and requires adjustments to provide a reliable quality assessment. Notably, it does not explicitly address important sources of bias, such as confounding and selective reporting [67], nor does it account for the accuracy of exposure assessment or the consideration of exposure–response relationships. In addition to the already discussed inadequate exposure assessment, another limitation was the lack of information in primary studies on potential confounders such as tobacco smoking, alcohol drinking, overweight or obesity, sedentary lifestyle, and dietary habits. To obscure an association between benzene exposure and risk of these cancer, however, these factors would need to have a favorable distribution among benzene‐exposed workers (e.g., lower prevalence of smoking or overweight among the exposed) which is not supported by existing literature [68, 69] or has not been adequately studied. Furthermore, since most studies involved industries with co‐exposure to other known carcinogens such as PAHs and butadiene, even if we did not find a higher risk of male genital cancers, we cannot exclude this bias in our estimates. Finally, only a small number of available studies were conducted outside North America and Europe, thus reducing the generalizability of our findings, in particular to countries with lower incidence and mortality from prostate cancer.

In conclusion, this study provides no clear evidence of an association between occupational exposure to benzene and the risk of male genital cancers. However, the interpretation of these findings should be tempered by the methodological limitations present in available literature. Notably, findings from subgroup analyses of higher‐quality studies suggest an increased risk for prostate cancer, highlighting the need for further methodologically sound studies to draw more definitive conclusions on the potential carcinogenic effects of benzene on the male genital tract.

## Author Contributions

**Paolo Boffetta, Monireh Sadat Seyyedsalehi,** and **Alessandro Godono:** conceptualization. **Monireh Sadat Seyyedsalehi, Alessandro Godono,** and **Maria Vittoria Picciaiola:** methodology. **Alessandro Godono** and **Monireh Sadat Seyyedsalehi:** data curation. **Monireh Sadat Seyyedsalehi, Maria Vittoria Picciaiola,** and **Paolo Boffetta:** statistical analysis. **Alessandro Godono, Monireh Sadat Seyyedsalehi, Andrea Quattrocolo,** and **Roberta Caradonna:** validation. **Alessandro Godono, Monireh Sadat Seyyedsalehi, Andrea Quattrocolo, Roberta Caradonna,** and **Maria Vittoria Picciaiola:** writing. **Monireh Sadat Seyyedsalehi** and **Boffetta:** reviewing and editing. **Alessandro Godono, Monireh Sadat Seyyedsalehi,** and **Paolo Boffetta:** supervision.

## Conflicts of Interest

PB has served as an expert witness for both plaintiffs and defendants in benzene litigation, not related to this study. The other authors declare no conflicts of interest.

## Supporting information

Supplementary Tables.

## Data Availability

The data that support the findings of this study are available on request from the corresponding author. The data are not publicly available due to privacy or ethical restrictions.
