# Supplementary material for: Occupational Benzene Exposure and Risk of Male Genital Cancers: A Systematic Review and Meta‐Analysis
Source: Am J Ind Med. 2025 May 27;68(8):666–78. doi: 10.1002/ajim.23740 (PMC12242111; doi:10.1002/ajim.23740)
Supplement: Supplementary file 1 — Supplementary Tables. [file AJIM-68-666-s001.docx]

**Supplementary tables**

| **Supplementary table 1 a,b** | PRISMA Checklist |
| --- | --- |
| **Supplementary figure 1** | Bubble plot of results on the association between benzene exposure and male genital cancer according to employment duration, follow up duration and quality score |
| **Supplementary table 2** | Detailed search strategy used on different databases (30th April 2024) |
| **Supplementary table 3** | PECOS Criteria for Inclusion and Exclusion of Studies |
| **Supplementary table 4** | Modifiable version of NEWCASTLE - OTTAWA QUALITY ASSESSMENT |
| **Supplementary table 5** | The results of Quality Assessment evaluated using modifiable NEWCASTLE - OTTAWA QUALITY ASSESSMENT SCALE |
| **Supplementary table 6** | Summary of GRADE ratings, with rationales. |

**Supplementary Table 1a. PRISMA Checklist**

| **Section and Topic** | **Item #** | **Checklist item** | **Location where item is reported** | |  |
| --- | --- | --- | --- | --- | --- |
| **TITLE** | | | |  | |
| Title | 1 | Identify the report as a systematic review. | P1 | |  |
| **ABSTRACT** | | | |  | |
| Abstract | 2 | See the PRISMA 2020 for Abstracts checklist. | P2 | |  |
| **INTRODUCTION** | | | |  | |
| Rationale | 3 | Describe the rationale for the review in the context of existing knowledge. | P3 | |  |
| Objectives | 4 | Provide an explicit statement of the objective(s) or question(s) the review addresses. | P3 | |  |
| **METHODS** | | | |  | |
| Eligibility criteria | 5 | Specify the inclusion and exclusion criteria for the review and how studies were grouped for the syntheses. | P4 | |  |
| Information sources | 6 | Specify all databases, registers, websites, organisations, reference lists and other sources searched or consulted to identify studies. Specify the date when each source was last searched or consulted. | P4 | |  |
| Search strategy | 7 | Present the full search strategies for all databases, registers and websites, including any filters and limits used. | P4 | |  |
| Selection process | 8 | Specify the methods used to decide whether a study met the inclusion criteria of the review, including how many reviewers screened each record and each report retrieved, whether they worked independently, and if applicable, details of automation tools used in the process. | P4 | |  |
| Data collection process | 9 | Specify the methods used to collect data from reports, including how many reviewers collected data from each report, whether they worked independently, any processes for obtaining or confirming data from study investigators, and if applicable, details of automation tools used in the process. | P4 | |  |
| Data items | 10a | List and define all outcomes for which data were sought. Specify whether all results that were compatible with each outcome domain in each study were sought (e.g. for all measures, time points, analyses), and if not, the methods used to decide which results to collect. | P4-5 | |  |
|  | 10b | List and define all other variables for which data were sought (e.g. participant and intervention characteristics, funding sources). Describe any assumptions made about any missing or unclear information. | P4-5 | |  |
| Study risk of bias assessment | 11 | Specify the methods used to assess risk of bias in the included studies, including details of the tool(s) used, how many reviewers assessed each study and whether they worked independently, and if applicable, details of automation tools used in the process. | P5 | |  |
| Effect measures | 12 | Specify for each outcome the effect measure(s) (e.g. risk ratio, mean difference) used in the synthesis or presentation of results. | P5 | |  |
| Synthesis methods | 13a | Describe the processes used to decide which studies were eligible for each synthesis (e.g. tabulating the study intervention characteristics and comparing against the planned groups for each synthesis (item #5)). | P5 | |  |
|  | 13b | Describe any methods required to prepare the data for presentation or synthesis, such as handling of missing summary statistics, or data conversions. | P5 | |  |
|  | 13c | Describe any methods used to tabulate or visually display results of individual studies and syntheses. | P5 | |  |
|  | 13d | Describe any methods used to synthesize results and provide a rationale for the choice(s). If meta-analysis was performed, describe the model(s), method(s) to identify the presence and extent of statistical heterogeneity, and software package(s) used. | P5 | |  |
|  | 13e | Describe any methods used to explore possible causes of heterogeneity among study results (e.g. subgroup analysis, meta-regression). | P5 | |  |
|  | 13f | Describe any sensitivity analyses conducted to assess robustness of the synthesized results. | P5 | |  |
| Reporting bias assessment | 14 | Describe any methods used to assess risk of bias due to missing results in a synthesis (arising from reporting biases). | P5 | |  |
| Certainty assessment | 15 | Describe any methods used to assess certainty (or confidence) in the body of evidence for an outcome. | P5 | |  |
| **RESULTS** | | | |  | |
| Study selection | 16a | Describe the results of the search and selection process, from the number of records identified in the search to the number of studies included in the review, ideally using a flow diagram. | P6,13 | |  |
|  | 16b | Cite studies that might appear to meet the inclusion criteria, but which were excluded, and explain why they were excluded. | P13 | |  |
| Study characteristics | 17 | Cite each included study and present its characteristics. | P6 | |  |
| Risk of bias in studies | 18 | Present assessments of risk of bias for each included study. | P6 | |  |
| Results of individual studies | 19 | For all outcomes, present, for each study: (a) summary statistics for each group (where appropriate) and (b) an effect estimates and its precision (e.g. confidence/credible interval), ideally using structured tables or plots. | P6 | |  |
| Results of syntheses | 20a | For each synthesis, briefly summarise the characteristics and risk of bias among contributing studies. | P6 | |  |
|  | 20b | Present results of all statistical syntheses conducted. If meta-analysis was done, present for each the summary estimate and its precision (e.g. confidence/credible interval) and measures of statistical heterogeneity. If comparing groups, describe the direction of the effect. | P6 | |  |
|  | 20c | Present results of all investigations of possible causes of heterogeneity among study results. | P6 | |  |
|  | 20d | Present results of all sensitivity analyses conducted to assess the robustness of the synthesized results. | P6 | |  |
| Reporting biases | 21 | Present assessments of risk of bias due to missing results (arising from reporting biases) for each synthesis assessed. | P6 | |  |
| Certainty of evidence | 22 | Present assessments of certainty (or confidence) in the body of evidence for each outcome assessed. | P6 | |  |
| **DISCUSSION** | | | |  | |
| Discussion | 23a | Provide a general interpretation of the results in the context of other evidence. | P7 | |  |
|  | 23b | Discuss any limitations of the evidence included in the review. | P8 | |  |
|  | 23c | Discuss any limitations of the review processes used. | P8 | |  |
|  | 23d | Discuss implications of the results for practice, policy, and future research. | P9 | |  |
| **OTHER INFORMATION** | | | |  | |
| Registration and protocol | 24a | Provide registration information for the review, including register name and registration number, or state that the review was not registered. | P4 | |  |
|  | 24b | Indicate where the review protocol can be accessed, or state that a protocol was not prepared. | NA | |  |
|  | 24c | Describe and explain any amendments to information provided at registration or in the protocol. | NA | |  |
| Support | 25 | Describe sources of financial or non-financial support for the review, and the role of the funders or sponsors in the review. | P9 | |  |
| Competing interests | 26 | Declare any competing interests of review authors. | P9 | |  |
| Availability of data, code and other materials | 27 | Report which of the following are publicly available and where they can be found: template data collection forms; data extracted from included studies; data used for all analyses; analytic code; any other materials used in the review. | NA | |  |

**Supplementary Table 1b. PRISMA Abstract Checklist**

| **Section and Topic** | **Item #** | **Checklist item** | **Reported (Yes/No)** |
| --- | --- | --- | --- |
| **TITLE** | | |  |
| Title | 1 | Identify the report as a systematic review. | Yes |
| **BACKGROUND** | | |  |
| Objectives | 2 | Provide an explicit statement of the main objective(s) or question(s) the review addresses. | Yes |
| **METHODS** | | |  |
| Eligibility criteria | 3 | Specify the inclusion and exclusion criteria for the review. | Yes |
| Information sources | 4 | Specify the information sources (e.g. databases, registers) used to identify studies and the date when each was last searched. | Yes |
| Risk of bias | 5 | Specify the methods used to assess risk of bias in the included studies. | Yes |
| Synthesis of results | 6 | Specify the methods used to present and synthesise results. | Yes |
| **RESULTS** | | |  |
| Included studies | 7 | Give the total number of included studies and participants and summarise relevant characteristics of studies. | Yes |
| Synthesis of results | 8 | Present results for main outcomes, preferably indicating the number of included studies and participants for each. If meta-analysis was done, report the summary estimate and confidence/credible interval. If comparing groups, indicate the direction of the effect (i.e. which group is favoured). | Yes |
| **DISCUSSION** | | |  |
| Limitations of evidence | 9 | Provide a brief summary of the limitations of the evidence included in the review (e.g. study risk of bias, inconsistency and imprecision). | Yes |
| Interpretation | 10 | Provide a general interpretation of the results and important implications. | Yes |
| **OTHER** | | |  |
| Funding | 11 | Specify the primary source of funding for the review. | Yes |
| Registration | 12 | Provide the register name and registration number. | Yes |

**Supplementary Figure 1:** Bubble plot of results on the association between benzene exposure and male genital cancer according to employment duration, follow up duration and quality score


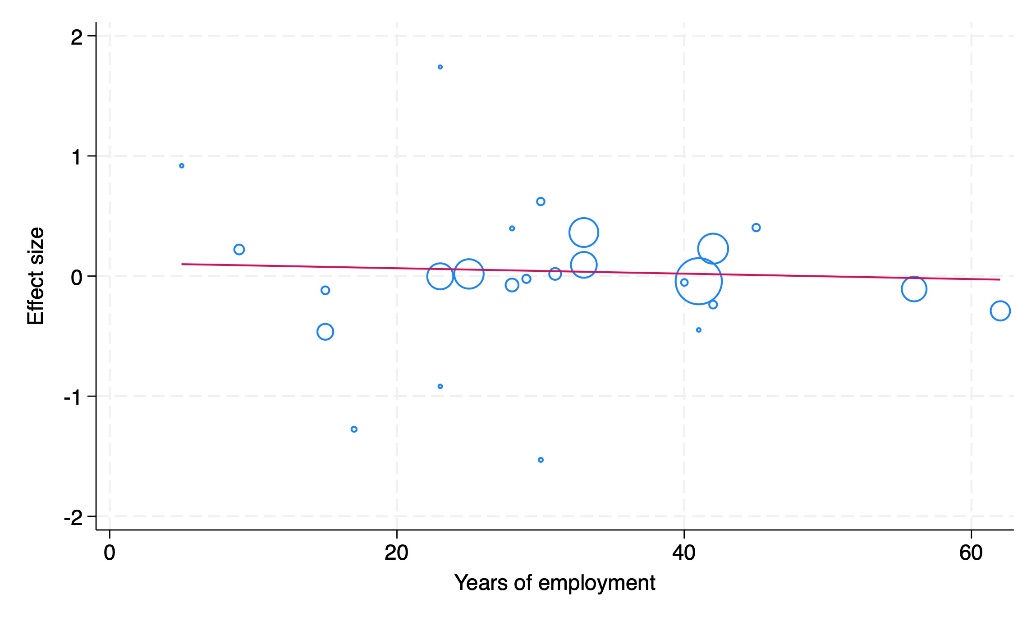


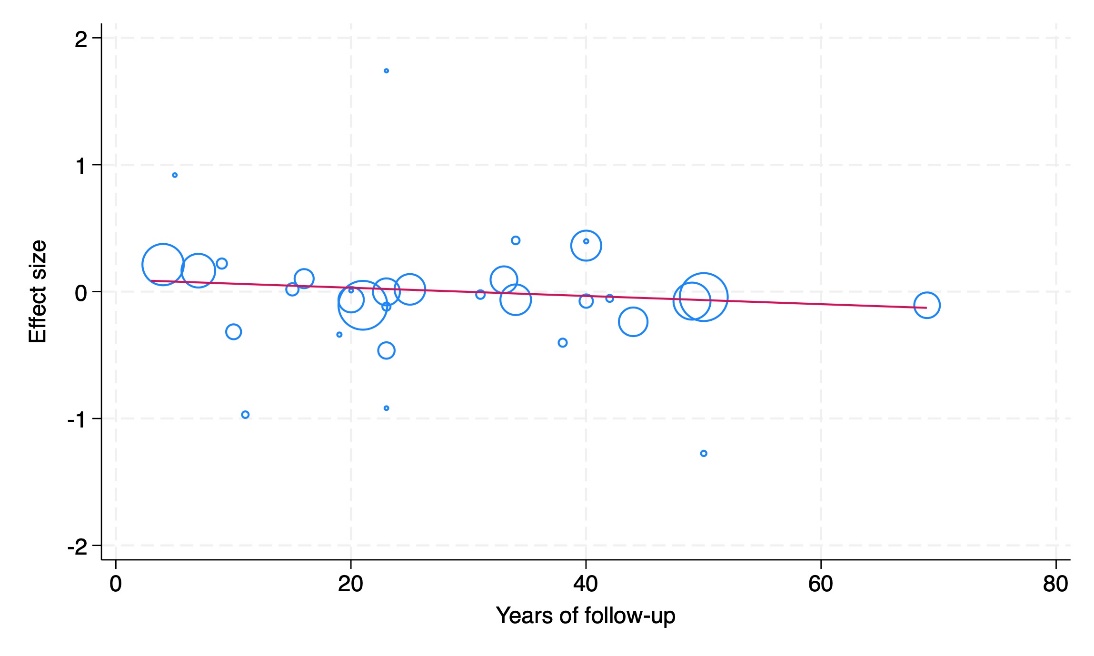

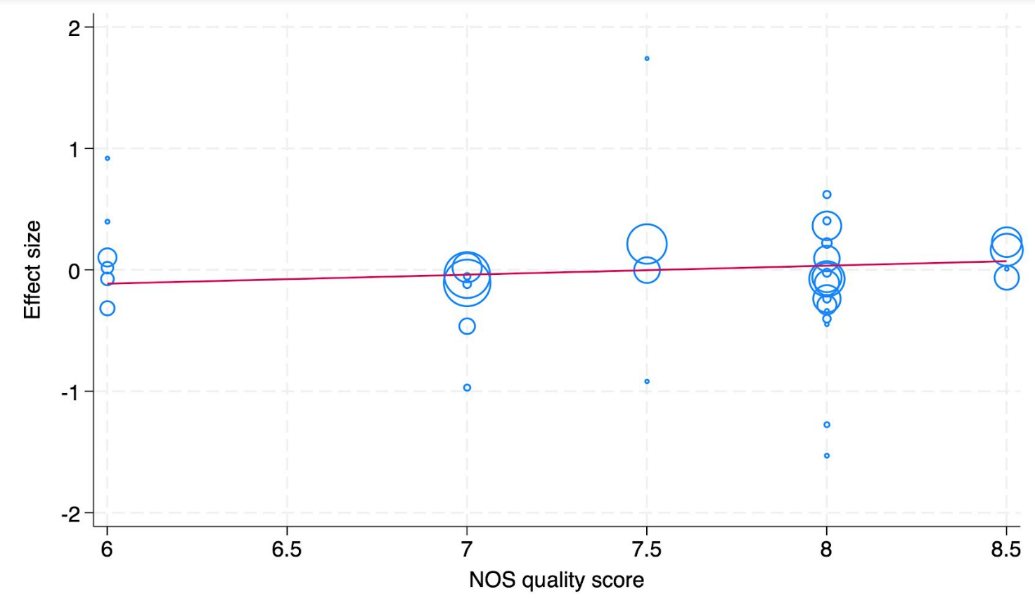

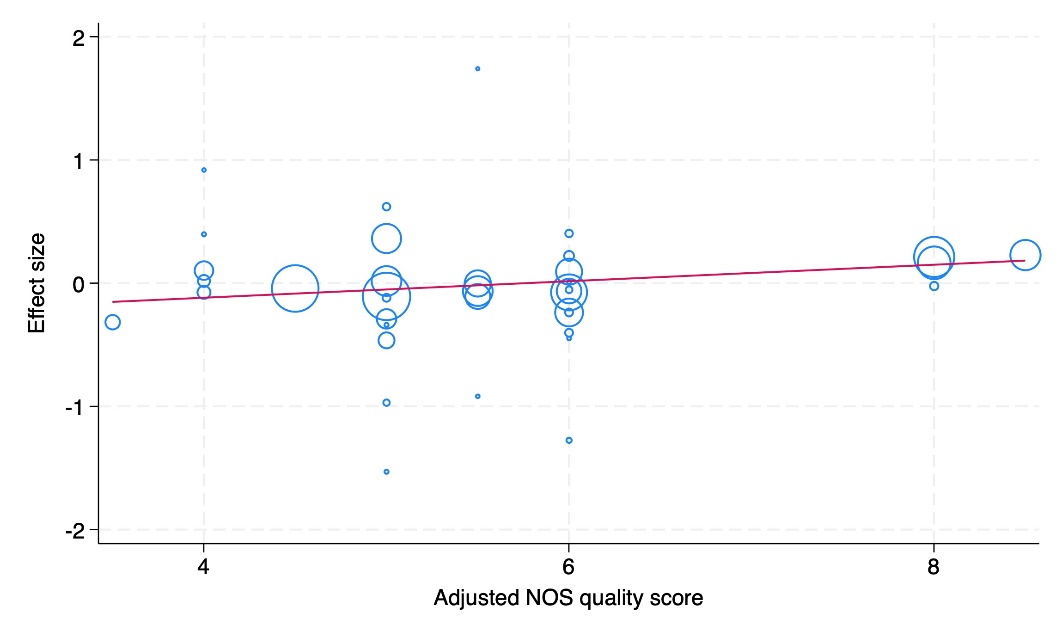


**Supplementary Table 2. Detailed search strategy used on different databases (30th April 2024)**

| **Database** | **Search string** |
| --- | --- |
| PubMed | (("neoplasms"[Title/Abstract] OR "carcinoma"[Title/Abstract] OR "cancer"[Title/Abstract] OR "malignant"[Title/Abstract]) AND ("benzene"[Title/Abstract] OR "benzol"[Title/Abstract] OR ("cyclohexa-1"[All Fields] AND "3 5 triene"[Title/Abstract]) OR (("1"[All Fields] AND "3"[All Fields]) AND "5-cyclohexatriene"[Title/Abstract]) OR "cyclohexatriene"[Title/Abstract])) AND ((humans[Filter]) AND (english[Filter] OR french[Filter] OR german[Filter] OR italian[Filter] OR spanish[Filter])) |
| Embase (Ovid) | ("benzene" or "'benzol" or "cyclohexa-1,3,5-triene" or "1,3,5-cyclohexatriene" or "cyclohexatriene").tw. and ("neoplasms" or "carcinoma" or "cancer" or "malignant").tw. limit to ((behavioral & social sciences or clinical medicine or health professions or life sciences or medical humanities or nursing or patient education or public health or science) and original articles) |
| Scopus | (( TITLE-ABS-KEY ( benzene ) OR TITLE-ABS-KEY ( benzol ) OR TITLE-ABS-KEY ( cyclohexa-1,3,5-triene ) OR TITLE-ABS-KEY ( 1,3,5-cyclohexatriene ) OR TITLE-ABS-KEY ( cyclohexatriene ))) AND (( TITLE-ABS-KEY ( neoplasms ) OR TITLE-ABS-KEY ( carcinoma ) OR TITLE-ABS-KEY ( cancer ) OR TITLE-ABS-KEY ( malignant ))) AND ( LIMIT-TO ( DOCTYPE , "ar" ) OR LIMIT-TO ( DOCTYPE , "re" )) AND ( LIMIT-TO ( SUBJAREA , "MEDI" ) OR LIMIT-TO ( SUBJAREA , "ENVI" )) AND ( LIMIT-TO ( LANGUAGE , "English" ) OR LIMIT-TO ( LANGUAGE , "German" ) OR LIMIT-TO ( LANGUAGE , "Italian" ) OR LIMIT-TO ( LANGUAGE , "French" ) OR LIMIT-TO ( LANGUAGE , "Spanish" )) AND ( LIMIT-TO ( SRCTYPE , "j" )) AND ( EXCLUDE ( SUBJAREA , "BIOC" ) OR EXCLUDE ( SUBJAREA , "EART" ) OR EXCLUDE ( SUBJAREA , "ENGI" ) OR EXCLUDE ( SUBJAREA , "CENG" )) AND ( EXCLUDE ( SUBJAREA , "COMP" ) OR EXCLUDE ( SUBJAREA , "MATH" )) AND ( EXCLUDE ( LANGUAGE , "Portuguese" ) OR EXCLUDE ( LANGUAGE , "Turkish" )) |

**Supplementary Table 3: PECOS Criteria for Inclusion and Exclusion of Studies**

|  | **INCLUSION CRITERIA** | **EXCLUSION CRITERIA** |
| --- | --- | --- |
| **Population** | Studies considering workers of all genders and ethnicities | Studies involving animals, blood, tissue or genetic biomarker |
| **Exposure** | Studies considering occupations where benzene is a significant/predominant source of exposure | Studies considering workers mainly exposed to other carcinogenic agents such as PAH, silica and butadiene |
| **Comparison** | Studies considering workers not exposed to benzene or external population comparison | None |
| **Outcome** | Studies reporting any risk measure of male genital cancers with their 95% CI or sufficient data for its computation | Studies on non-solid tumors (e.g., lymphoma, leukemia, myeloma) |
| **Study Design** | Observational studies presenting nested case-control and cohort study designs | Every study with a study design other than cohort or nested case-control |

**Supplementary Table 4 (a and b):** Modifiable version of NEWCASTLE - OTTAWA QUALITY ASSESSMENT SCALE (4a Case Control studies; 4b Control Studies)

**4A. CASE CONTROL STUDIES** (maximum score: 9)

Note: A study can be awarded a maximum of one star for each numbered item within the Selection and Exposure categories. A maximum of two stars can be given for Comparability.

**Selection**

**1) Is the case definition adequate?**

a) yes, with independent validation **(1)**

b) yes, eg record linkage **(1)** or based on self-reports **(0.5)**

c) no description **(0)**

**2) Representativeness of the cases**

a) consecutive or obviously representative series of cases **(1)**

b) potential for selection biases or not stated **(0)**

**3) Selection of Controls**

a) community controls **(1)**

b) hospital controls **(0.5)**

c) no description **(0)**

**4) Definition of Controls**

a) no history of disease (endpoint) **(1)**

b) no description of source **(0)**

**Comparability**

**1) Comparability of cases and controls on the basis of the design or analysis**

a) study controls for age, gender, province **(0)**

b) study controls for age, gender, province +smoking **(1)**

c) study controls for age, gender, province +smoking + other additional factors **(2)**

**Exposure**

**1) Ascertainment of exposure**

a) secure record (eg surgical records) **(1)**

b) structured interview where blind to case/control status **(1)**

c) interview not blinded to case/control status **(0.5)**

d) written self-report or medical record only **(0.5)**

e) no description **(0)**

**2) Same method of ascertainment for cases and controls**

a) yes **(1)**

b) no **(0)**

**3) Non-Response rate**

a) one or both groups over 90% **(1)**

b) one or both groups between 60- 90% **(0.5)**

c) one or both groups under 60% **(0)**

d) no statemen **(0)**

NEWCASTLE - OTTAWA QUALITY ASSESSMENT SCALE

**4B. COHORT STUDIES** (maximum score: 10)

Note: A study can be awarded a maximum of one star for each numbered item within the Selection and

Outcome categories. A maximum of two stars can be given for Comparability.

**Selection**

**1) Representativeness of the exposed cohort**

a) truly representative of the average _______________ (describe) in the community **(2)**

b) somewhat representative of the average ______________ in the community **(1)**

c) selected group of users eg nurses, volunteers **(0.5)**

d) no description of the derivation of the cohort **(0)**

**2) Selection of the non-exposed cohort**

a) drawn from the same community as the exposed cohort **(1)**

b) drawn from a different source **(0.5)**

c) no description of the derivation of the non-exposed cohort **(0)**

**3) Ascertainment of exposure**

a) secure record (eg surgical records) **(1)**

b) structured interview **(1)**

c) written self-report **(0.5)**

d) no description **(0)**

**4) Demonstration that outcome of interest was not present at start of study**

a) yes **(1)**

b) no **(0)**

**Comparability**

**1) Comparability of cohorts on the basis of the design or analysis**

a) study controls for age, gender, province **(0)**

b) study controls for age, gender, province +smoking **(1)**

c) study controls for age, gender, province +smoking + other additional factors **(2)**

**Outcome**

**1) Assessment of outcome**

a) independent blind assessment **(1)**

b) record linkage **(1)**

c) self-report **(0.5)**

d) no description **(0)**

**2) Was follow-up long enough for outcomes to occur**

a) yes (select an adequate follow up period for outcome of interest) **(1) (average 15 years)**

b) no **(0)**

**3) Adequacy of follow up of cohorts**

a) complete follow up - all subjects accounted for over 90% **(1)**

b) subjects lost to follow up unlikely to introduce bias - small number lost - > ____ % (select an

adequate %) follow up, or description provided of those lost) between 60- 90% **(0.5)**

c) follow up rate < ____% (select an adequate %) and no description of those lost under 60% **(0)**

d) no statemen **(0)**

**Supplementary table 5: The results of Quality Assessment evaluated using modifiable NEWCASTLE - OTTAWA QUALITY ASSESSMENT SCALE**

**5a) CASE CONTROL STUDIES**

| **First Author** | **Q1**  **Selection** | **Q2**  **Selection** | **Q3**  **Selection** | **Q4**  **Selection** | **Q1**  **Comparability** | **Q1 Exposure** | **Q2 Exposure** | **Q3 Exposure** | **Total Score** | **Dose**  **(0 vs 2)** | **Adjusted score** |
| --- | --- | --- | --- | --- | --- | --- | --- | --- | --- | --- | --- |
| Greenland S | 1 | 1 | 1 | 1 | 0 | 1 | 1 | 0 | 6 | -2 | 4 |
| Gérin M | 1 | 1 | 1 | 1 | 2 | 0.5 | 1 | 1 | 8 | 0 | 8.5 |
| Krishnadasan A | 1 | 1 | 1 | 1 | 2 | 1 | 1 | 0,5 | 8,5 | 0 | 8 |
| Blanc-Lapierre A | 1 | 1 | 1 | 1 | 2 | 0.5 | 1 | 0 | 8 | 0 | 8 |
| Wilcosky TC | 1 | 1 | 1 | 1 | 0 | 0.5 | 1 | 0 | 5.5 | -2 | 3.5 |

**Supplementary table 5: The results of Quality Assessment evaluated using modifiable NEWCASTLE - OTTAWA QUALITY ASSESSMENT SCALE**

**5b) COHORT STUDIES**

| **First Author** | **Q1**  **Selection** | **Q2**  **Selection** | **Q3**  **Selection** | **Q4**  **Selection** | **Q1**  **Comparability** | **Q1 Outcome** | **Q2 Outcome** | **Q3 Outcome** | **Total Score** | **Dose**  **(0 vs 2)** | **Adjusted score** |
| --- | --- | --- | --- | --- | --- | --- | --- | --- | --- | --- | --- |
| Ott MG | 2 | 1 | 1 | 1 | 0 | 1 | 1 | 0 | 7 | -2 | 5 |
| Rushton L | 2 | 0.5 | 0.5 | 1 | 1 | 1 | 1 | 0 | 7 | -2 | 5 |
| Guberan E | 2 | 1 | 1 | 1 | 0 | 1 | 1 | 1 | 8 | -2 | 6 |
| Bond GG | 2 | 1 | 1 | 1 | 0 | 1 | 1 | 1 | 8 | -2 | 6 |
| Wong O | 2 | 1 | 1 | 1 | 0 | 1 | 1 | 1 | 8 | 0 | 8 |
| Paci E | 2 | 1 | 1 | 1 | 0 | 1 | 1 | 1 | 8 | -2 | 6 |
| Wongsrichanalai C. | 2 | 1 | 1 | 1 | 0 | 1 | 1 | 1 | 8 | -2 | 6 |
| Szeszenia-D N | 2 | 1 | 1 | 1 | 0 | 0 | 1 | 0 | 6 | -2 | 4 |
| Lagorio S | 2 | 1 | 0.5 | 1 | 0 | 1 | 1 | 0.5 | 7 | -2 | 5 |
| Honda Y | 2 | 1 | 1 | 1 | 0 | 1 | 1 | 1 | 8 | -2 | 6 |
| Satin K.P. | 2 | 1 | 0.5 | 1 | 0 | 1 | 1 | 0 | 6.5 | -2 | 4.5 |
| Collingwood KW | 2 | 1 | 0 | 1 | 0 | 1 | 1 | 1 | 7 | -2 | 5 |
| Järvholm B | 2 | 1 | 1 | 1 | 0 | 1 | 1 | 1 | 8 | -2 | 6 |
| Lynge E | 2 | 0.5 | 0.5 | 1 | 0 | 1 | 1 | 1 | 7 | -2 | 5 |
| Pukkala, E | 2 | 1 | 1 | 1 | 0 | 1 | 1 | 0 | 7 | -2 | 5 |
| Consonni D. | 2 | 1 | 1 | 1 | 0 | 1 | 1 | 1 | 8 | -2 | 6 |
| Kauppinen T | 2 | 1 | 1 | 1 | 0 | 1 | 1 | 1 | 8 | -2 | 6 |
| Lewis, R J | 2 | 1 | 1 | 1 | 0 | 1 | 1 | 0 | 7 | -2 | 5 |
| Sorahan T | 2 | 1 | 0.5 | 1 | 0 | 1 | 1 | 1 | 7.5 | -2 | 5.5 |
| Swaen G. | 2 | 1 | 1 | 1 | 0 | 1 | 1 | 1 | 8 | -2 | 6 |
| Gun RT | 2 | 0.5 | 0.5 | 1 | 1 | 1 | 1 | 1 | 8 | -2 | 6 |
| Budroni M | 1 | 1 | 1 | 1 | 0 | 1 | 1 | 0 | 6 | -2 | 4 |
| Bonneterre V | 2 | 1 | 1 | 1 | 0 | 1 | 1 | 0.5 | 7.5 | -2 | 5.5 |
| Koh DH | 2 | 1 | 1 | 1 | 0 | 1 | 0 | 0 | 6 | -2 | 4 |
| Collins J. | 2 | 1 | 0.5 | 1 | 0 | 1 | 1 | 1 | 7.5 | -2 | 5.5 |
| Bonzini M | 2 | 1 | 0 | 1 | 0 | 1 | 1 | 1 | 7 | -2 | 5 |

**Supplementary table 6**. Summary of GRADE ratings, with rationales.

|  | **Overall Risk of Prostate Cancer** | | **Risk of Mortality of Prostate Cancer** | | **Risk of Incidence of Prostate Cancer** | |
| --- | --- | --- | --- | --- | --- | --- |
|  | **Rating** | **Rationale** | **Rating** | **Rationale** | Rating | **Rationale** |
| **Risk of Bias** | - 1 | The rating was downgraded by one point due to a significant risk of bias in much of the available evidence. Most studies (25 out of 29) showed a substantial risk of confounding bias. Additionally, some studies had insufficient follow-up periods (9 out of 29), further heightening the concern about bias. | -1 | The rating was downgraded by one point due to a significant risk of bias in much of the available evidence. A substantial risk of confounding bias was present in most studies (19/21). Moreover 5 out of 21 studies didn't have an adequate follow-up period for the outcome of interest. | -1 | The rating was downgraded by one point because the majority of evidence came from studies with a high risk of confounding bias (11/14 studies). |
| **Indirectness** | 0 | The rating was not downgraded because there is no compelling reason to believe that the studied population differed significantly from the population of interest in a way that would impact effect sizes. Moreover, the included studies were coherent with our research question. | 0 | The rating was not downgraded because there is no strong reason to believe that the studied population differed from the population of interest in a manner that would affect effect sizes. No study was considered at risk of selection bias. | 0 | The rating was not downgraded because there is no indication that the studied population differed significantly from the population of interest in a way that would influence effect sizes. Additionally, none of the studies were deemed to be at risk of selection bias. |
| **Inconsistency** | 0 | The rating was not downgraded since the overall I^2^ indicated low heterogeneity, the test of heterogeneity was not significant, and the confidence intervals were overlapping. | 0 | The rating was not downgraded because the heterogeneity captured by I^2^ was equal to 0, the p-value for the heterogeneity test wasn’t significant and the confidence intervals were overlapping. | 0 | The rating was not downgraded because the heterogeneity captured by I^2^ showed a low level of heterogeneity in the results and most of the point estimates were consistent. |
| **Imprecision** | 0 | The rating was not downgraded due to imprecision since the confidence interval around the estimate of exposure was sufficiently narrow. | 0 | The rating was not downgraded due to imprecision since the confidence interval around the estimate of exposure was sufficiently narrow. | 0 | The rating was not downgraded due to imprecision since the confidence interval around the estimate of exposure was sufficiently narrow. |
| **Publication bias** | 0 | The rating was not downgraded due to publication bias because the visual inspection of the funnel did not indicate concerning evidence of asymmetry, and the p-value of the Egger test was not significant (p=0.117). | 0 | The rating was not downgraded because the visual inspection of the funnel plot didn’t show any concerning evidence of publication bias and the p-value of the Egger test was not significant. | 0 | The rating was not downgraded due to publication bias because the visual inspection of the funnel did not indicate concerning evidence of asymmetry, and the p-value of the Egger test was not significant. |
| **Large magnitude** | 0 | The rating was not upgraded because the pooled estimate was not significant. | 0 | The rating was not upgraded because the magnitude of the effect did not demonstrate a strong association between the outcome and exposure. | 0 | The rating was not upgraded because the effect size did not indicate a strong association between the outcome and exposure. |
| **Exposure - response** | 0 | The rating was not upgraded since exposure response gradients were not available across most of the studies included. | 0 | The rating was not upgraded since exposure response gradients were not available across the majority of the studies included. | 0 | The rating was not upgraded since exposure response gradients were not available across the majority of the studies included. |
| **Residual Confounding** | 0 | The rating was not upgraded because plausible residual confounding is likely to have minimal impact on the estimates. | 0 | The rating was not increased because the analysis suggests that any potential residual confounding (such as smoking or lifestyle factors) would likely attenuate the observed association. This means that the true association could be even weaker than observed in the presence of unmeasured residual confounding. | 0 | The rating was not increased because the analysis indicates that any potential residual confounding would likely diminish the observed association, biasing the results toward the null. This suggests that the true association may be even weaker than what was observed, given the presence of unmeasured residual confounding. |

|  | **Overall Risk of Testis Cancer** | | **Risk of Mortality of Testis Cancer** | | **Risk of Incidence of Testis Cancer** | |
| --- | --- | --- | --- | --- | --- | --- |
|  | **Rating** | **Rationale** | **Rating** | **Rationale** | **Rating** | **Rationale** |
| **Risk of Bias** | -1 | The rating was downgraded by one point due to a significant risk of bias in all the included studies. All studies presented high risk of confounding bias. | -1 | The rating was downgraded by one point due to a significant risk of bias in all the included studies. All studies presented high risk of confounding bias. | -1 | The rating was downgraded by one point because all studies presented a high risk of confounding bias. All studies presented high risk of confounding bias. |
| **Indirectness** | 0 | The rating was not downgraded because there is no strong reason to believe that the studied population differed from the population of interest in a manner that would affect effect sizes. The studies included were not at risk of selection bias and they were coherent with our research question. | 0 | The rating was not downgraded because there is no strong reason to believe that the studied population differed from the population of interest in a manner that would affect effect sizes. The studies included were not at risk of selection bias and they were coherent with our research question. | 0 | The rating was not downgraded because there is no indication that the studied population differed significantly from the population of interest in a way that would influence effect sizes. All included studies addressed our research question coherently. |
| **Inconsistency** | 0 | The rating was not downgraded because the heterogeneity captured by I^2^ was equal to 0, the p-value for the heterogeneity test wasn’t significant and the confidence intervals were overlapping. | 0 | The rating was not downgraded because the heterogeneity captured by I^2^ was equal to 0, the p-value for the heterogeneity test wasn’t significant and the confidence intervals were overlapping. | 0 | The rating was not downgraded because the heterogeneity captured by I^2^ was equal to 0, the p-value for the heterogeneity test wasn’t significant and the confidence intervals were overlapping. |
| **Imprecision** | 0 | The rating was not downgraded due to imprecision because the c onfidence interval around the estimate of exposure was sufficiently narrow. | -1 | The rating was downgraded due to imprecision because the confidence interval around the exposure estimate was wide, thus compromising the reliability of the estimate and reducing the overall quality of the evidence. | 0 | The rating was not downgraded due to imprecision because the c onfidence interval around the estimate of exposure was sufficiently narrow. |
| **Publication bias** | 0 | The rating was not downgraded because the visual inspection of the funnel and DOI plots didn’t show any concerning evidence of publication bias and the p-value of the Egger test was not significant (p=0.301). | 0 | The rating was not downgraded because the visual inspection of the funnel and DOI plots didn’t show any concerning evidence of publication bias and the p-value of the Egger test was not significant (p=0.201). | 0 | The rating was not downgraded due to publication bias because the visual inspection of the funnel plot did not indicate any evidence of asymmetry, and the p-value of the Egger test was not significant (p=0.724) |
| **Large magnitude** | 0 | The rating was not upgraded because the pooled effect size was not significant. | 0 | The rating was not upgraded because the pooled effect size was not significant. | 0 | The rating was not upgraded because the effect size was not significant. |
| **Exposure response** | 0 | The rating was not upgraded since exposure response gradients were not available in the studies included. | 0 | The rating was not upgraded since exposure response gradients were not available in the studies included. | 0 | The rating was not upgraded since exposure response gradients were not available across the included studies. |
| **Residual Confounding** | 0 | The rating was not upgraded because plausible residual confounding is likely to have minimal impact on the estimates. | 0 | The rating was not upgraded because plausible residual confounding is likely to have minimal impact on the estimates. | 0 | The rating was not upgraded because plausible residual confounding is likely to have minimal impact on the estimates. |
